# NutriColoring: designing a doodling toolkit to support daily self-reported dietary assessment among office workers

**DOI:** 10.3389/fpsyg.2023.1265218

**Published:** 2023-12-07

**Authors:** Sibo Pan, Xipei Ren, Steven Vos, Aarnout Brombacher

**Affiliations:** ^1^Department of Industrial Design, Eindhoven University of Technology, Eindhoven, Netherlands; ^2^School of Design and Arts, Beijing Institute of Technology, Beijing, China; ^3^School of Sport Studies, Fontys University of Applied Sciences, Eindhoven, Netherlands

**Keywords:** healthy eating, office worker, dietary assessment, self-report, doodling, coloring

## Abstract

This study was motivated by a desire to help working-age individuals gain a better understanding of their daily nutritional intakes with a new self-reported dietary assessment method because an unhealthy eating behavior increases the risks of developing chronic diseases. In this study, we present the design and evaluation of NutriColoring, a food diary that leverages doodling on sketches to report and reflect on everyday diet in the working context. Through a 2-week field study involving 18 participants, the usefulness of NutriColoring in facilitating dietary assessment was tested by making comparisons with the typical bullet diary method. Our quantitative results showed that NutriColoring provided users with improved dietary assessment experience and intrinsic motivations, with significantly low task frustration and high enjoyment. Because of the freedom and playfulness in reporting intakes at work, the interview findings showed a high acceptance of employing NutriColoring at work. This article is concluded with a set of implications for the design and development of a Doodling toolkit to support healthy eating behaviors among office workers.

## 1 Introduction

The workplace context plays an essential role in influencing eating behaviors among office workers. A typical working-age adult spends up to two-thirds of their waking hours in the working context and consumes approximately a third of their daily food intake at work (Gorman et al., 2013). Given that unhealthy eating behaviors within the working context have been shown to be associated with increased risk of conditions such as diabetes, obesity, and heart diseases (Naessens et al., 2011), the promotion of healthy eating behaviors has been identified as a crucial determinant influencing individuals’ overall well-being and health (Hartline-Grafton et al., 2010; Lima et al., 2018). Moreover, after the COVID-19 pandemic, a shift toward remote working across diverse contexts (i.e., office and home office) has grown as a new working mode (Allen et al., 2015). This shift has contributed to unhealthy eating patterns, including increased consumption of unhealthy foods, larger portion sizes during main meals, and more snacks between meals (Ammar et al., 2020). Consequently, the demand for assistance in performing healthy eating activities and reporting daily intake among office workers is on the rise.

Self-reported dietary assessment tools have been increasingly developed (Maes et al., 2012; Hipp et al., 2015) because it has the potential to facilitate automated intake data collection and support analyses with data visualizations (Elsden et al., 2017). People engage in self-reporting as it helps them to develop specific self-awareness of healthy nutritional practices (Lazar et al., 2015; Elsden et al., 2016). Applying automated self-reporting assessment tools in a daily context, however, addresses several issues: (1) absence of personally meaningful insights (Epstein et al., 2016), (2) unmanageable maintenance (Harrison et al., 2015), (3) limited flexibility in reporting items for individual needs (Kim et al., 2017), and (4) technological boundaries (Ayobi et al., 2017). These issues partially led to abandon of digital reporting tools over time and a switch to paper notes to avoid unintended effects (Lazar et al., 2015; Epstein et al., 2016). Paper-based dietary tools are capable of mindful self-reporting practices (Ayobi et al., 2018). The usage of these papery tools can improve flexibility to construct self-reporting processes, satisfy realistic nutritional needs, and help to achieve personal eating goals (Lazar et al., 2015).

Based on user preference for self-reported tools identified in our prior research (Pan et al., 2021), office workers have a predilection for employing tangible supplies to facilitate a creative and playful self-reporting practice, rather than depending on mobile applications in their working contexts. In this regard, paper-based tools, such as Doodling, can afford physical practices such as writing, crafting, and sketching to engage users in reporting personal health status (Andrade, 2010). Doodling, as a beneficial and pleasurable tool for personal care to maintain overall health (Coward, 2023), could lower the threshold of self-reporting and increase the interest in reporting personal data (Fernandes et al., 2018; Meade et al., 2019). Recent studies have indicated that the Doodling tools might be advantageous in the daily working context as it is believed to keep focus on primary tasks without affecting attention or raising mind wandering during working hours (Andrade, 2010; Chan, 2012). However, the complex process underlying the decision to adopt or reject any given Doodling tools of dietary self-reporting practices in daily working contexts requires further exploration (Coward, 2023). Thus, we developed our first research question as follows:

RQ1: How can Doodling be designed and leveraged as a self-reported dietary assessment method for office workers?

Doodling is a creative method to draw and visualize ideas (Meade et al., 2019). Various modalities for Doodling tools designed to enhance the engagement of self-reporting have been studied extensively. Evidence indicates that the Doodling with coloring approach should set out to investigate potential health-related activities (Ashlock et al., 2018; Burton and Baxter, 2019; Xi et al., 2022) because using colors to visualize nutrition information could be an effective technique for increasing positive understanding of daily food intake (Ursell, 2007). For instance, Deanna (Minich, 2019) pointed out that the concept of Eat a rainbow (i.e., group fruits and vegetables according to their natural colors; people should consume each hue of fruits and vegetables to acquire a range of various vitamins and nutrients that can prevent eating-related diseases) helps people readily relate to the health properties of healthy intake (i.e., fruits and vegetables) and develop a strong sense of self-awareness through colors (Heber, 2004). However, Doodling via coloring approach was mostly explored for self-reporting behavioral and physiological anxiety (Ashlock et al., 2018; Burton and Baxter, 2019; Xi et al., 2022). In-depth studies directly examining how office workers perceive Doodling with coloring approach and react to its use in daily working context are limited (Elsden et al., 2016). Hence, it would be interesting to investigate the effects of a color-based Doodling tool within a working context. To this end, we develop the second research question as follows:

RQ2: Whether and how the developed Coloring-based Doodling method can help office workers engage in self-reporting on daily eating practices?

In this study, we present the design and evaluation of NutriColoring. NutriColoring is a Doodling toolkit with a coloring approach to promoting the self-reporting practice of daily intake in the working context. To examine the acceptance and intrinsic motivation of using the NutriColoring toolkit, we conducted a 2-week field study with 18 working-age individuals. The study was designed as a within-subject experiment, where we compared NutriColoring to a traditional food Journaling toolkit (named NutriWriting in this study). We collected and analyzed quantitative questionnaire data as well as qualitative interview data to gain a deep understanding of the user experience of the NutriColoring toolkit and then identify design opportunities for the subsequent development of the Doodling via coloring approach.

The remainder of this article is organized as follows. In the next section, we provide a review of related literature on Doodling and coloring approach. Then, in Section 3, we described the study method and material regarding the toolkits (i.e., NutriColoring and NutriWriting), study design, and data analysis. In Section 4, we reported both quantitative and qualitative results of our study, which led to a discussion on the findings and limitations, with implications for future study, in Section 5. Section 6 contains our conclusions.

## 2 Related study

In this section, we demonstrate two types of related studies. First, we give an overview of how self-reported tools are applied to promote healthy eating patterns. Second, we go into the Doodling approach with the use of colors for self-reporting practices, particularly for health and well-being.

### 2.1 Self-report for eating practice during office work

Self-report has been investigated in many fields, for instance, personal information management (Van Kleek et al., 2009), lifelogging (Sellen and Whittaker, 2010), personal informatics (Elsden et al., 2016), and applied design methods (Carter and Mankoff, 2005; Ayobi et al., 2018). It is an essentially human expressive practice that involves documenting and organizing daily experiences in an effort to beneficially stimulate health and well-being (Lepore and Smyth, 2002; Ayobi et al., 2018). Self-reporting for assessing food intake has been increasingly examined in the HCI research field (Cordeiro et al., 2015a,b). On the one hand, a convergence of a wide range of digital dietary assessment tools—such as Compl-eat™ (Meijboom et al., 2017), Traqq (Lucassen et al., 2021), and Dutch FFQ-TOOL™ (Molag, 2010)—has made it possible for people to obtain accurate data and receive pertinent feedback (Burke et al., 2005). On the other hand, dietary assessment tools have become a social approach (Lupton, 2014), while helping individuals gain self-awareness of daily intake (Kersten-van Dijk et al., 2017). For instance, Nour et al. (2019) used reward mechanisms and social media impacts in a self-reporting app to encourage more vegetable intake among young adults. Chung et al. (2017) indicated that sharing food pictures on Instagram could motivate peers and seek support for adaptive healthy eating behaviors as well as eating goals.

Several studies have investigated the barriers for users to adopting digital self-reported tools and suggested that digital tools might induce negative feelings or unintended effects, resulting in refusal to use these technologies in daily contexts (Lazar et al., 2015; Epstein et al., 2016). In a survey of the National Health in America (Tracking for Health | Pew Research Center, n.d.), it was found that 34% of users use pencil and paper, while 21% use digital technologies for daily self-reporting. The usage of paper-based dietary assessment tools shows a slightly higher adoption rate. The reasons for this situation could be the flexibility of reporting ways on paper, satisfying volatile eating needs, personalizing eating goals, etc. (Lazar et al., 2015). Also, failing to meet security and privacy requirements leads to choosing paper-based self-reported tools (Epstein et al., 2017).

Moreover, the acceptance of self-reported dietary assessment tools in the working context is an important emerging topic. According to Naska et al. (2017), self-reported dietary assessment can be roughly divided into two categories: prospective methods (i.e., food diary) and retrospective methods (e.g., dietary recall and food frequency questionnaires). The prior research (Pan et al., 2022) shows that the food diary ensured more flexible self-reporting for office workers to assess their daily intakes than retrospective assessment methods. A growing number of designs have considered facilitating self-report and daily nutrition tracking for the everyday context. For instance, MyFitnessPal (Byrne, 2015) supports healthy eating by relying on associating food ingredients with calories. Eat&Tell (Achananuparp et al., 2018) is designed to facilitate the collection of eating-related data through automated tracking and self-report. By scanning QR codes on food packages, other designs (Sysoeva et al., 2017; Hartwell et al., 2019) focused on encouraging healthy food choices and providing food-related feedback to users. Although these self-reported tools have focused on tracking food consumption and improving eating behaviors, less attention has been paid to promoting healthy eating patterns and routines in the working context. Also, despite the benefits of using a food diary for self-reporting, office workers are still lagging in terms of the utilization rate, and the need for a paper-based food diary has not translated into a long-term willingness to use it in daily working routines. There is much scope for considering self-reported tools in the context of the worksite.

### 2.2 Doodling as a self-reporting approach

According to the Oxford English Dictionary, Doodling is “a random scribbling performed by a person while the mind is more or less otherwise applied.” Furthermore, earlier studies intended to explore the advantages of doodling as a viable means of collecting notes and memory retention (Andrade, 2010). Doodling is also a common means and creative form, which has been shown to positively contribute to self-care and self-expression (Stuckey and Tisdell, 2010). Evidence supports that offering engaging ways for people to participate in the reporting process is one approach to promoting *self-care* (Coward, 2023). Self-care is the ability to actively take care of one’s mental, physical, and emotional health. For instance, Liu et al. (2020) explore new utilizations based on the idea of user doodles for communication and reporting of dietary. Their findings suggested that Doodling might be an enjoyable and effective form of self-care for people to engage in the nutrition and health domain. Prior research also stated that Doodling enables to lead to an improvement in a person’s behavior with ongoing reflection (Barnett and Cooper, 2009; Wallace, 2020), which may optimize self-awareness of personal health and overall sense of well-being.

In recent decades, Doodling has been increasingly popularized as a tool for *self-expression* through coloring books (Coward, 2023). According to prior research, doodling in art-making form (e.g., drawing and painting) could be a helpful reporting practice for long-term positive effects on health (Stuckey and Nobel, 2010). It gives not only the artist but also the normal population the ability to tell their individual stories visually and internally (Coward, 2023). Compared to telling stories with a text-based reporting approach (e.g., Journaling), Doodling in art form provides a significant improvement in using engagement (Kim and Sherman, 2007) and also plays as a positive psychological way for people to experience enjoyment during the reporting process (John, 2012). For instance, many recent studies (Clark and Dünser, 2012; Muthard and Gilbertson, 2016; Turturro and Drake, 2022) have shown that Doodling on a coloring book for adults was a beneficial medium for self-reporting states, especially for regulating negative feelings. Two types of coloring approaches were generally used for self-reporting, namely, well-designed coloring notebook (e.g., Mandala, a circle made up of various lined forms and patterns on a notebook) and free coloring activity (i.e., people are not given instructions on what to paint on the paper) (Mantzios and Giannou, 2018). Traditionally, it has been argued that there is no difference between these two approaches, but some studies (Curry and Kasser, 2005; Taylor, 2016) examined that a well-designed coloring Doodling was a more useful self-reporting form.

On the other hand, color plays a vital role in the food industry in triggering purchasing behaviors and creating important expectations regarding the flavor and visual appeal of food (Spence, 2016; Stich, 2016). Previous studies suggested that self-reporting via the coloring approach should not only focus on reducing negative affect (e.g., behavioral and physiological anxiety) but also should set out to investigate potential health-related perspectives (e.g., sedentary lifestyle and healthy eating) through personalization and customization (Ashlock et al., 2018; Burton and Baxter, 2019; Xi et al., 2022). According to Piqueras-Fiszman and Spence (2014), altering the color aspects related to food (e.g., the color of plateware/container and packaging; color of the context where foods are eaten) can modify people’s perception and motivation to choose healthy foods. Among interventions aimed at promoting healthier food choices, the Traffic Light Diet (red for unhealthy, yellow for less healthy, and green for healthy) was widely used in the mHealth domain. For instance, Turner-McGrievy et al. (2016) integrated a Traffic light diet to help participants reduce the burden of dietary self-monitoring and provide easy-to-understand feedback. Johnson et al. (2014) evaluated that a food recommendation system based on the Traffic light diet could give consumers tips for healthier food choices when dining out. Aschemann-Witzel et al. (2013) found that color-coding labels replacing literal labels could increase the consumption of nutritional products. Montagni et al. (2020) displayed green labels on healthy food items according to the Traffic light diet developed by Leonard et al. and NUTRI-SCORE (Julia et al., 2018) to increase healthy food intake in worksites. Regarding the dietary self-reporting approach with colors, the MyPlate (MyPlate | U.S. Department of Agriculture, n.d.) app was developed food categories into five, namely, fruits (in red), vegetables (in green), grains (in orange), protein (in purple), and dairy (in blue). This application enables users to report daily intake according to corresponding colors and then provides an overview of food consumption within colors and reminds the distance between users’ actual intake and balanced intake reference.

In summary, the evidence shows an excellent opportunity to deploy dietary self-reported tools in the context of the workplace. Compared to digital reporting tools, office workers prefer paper reporting tools to avoid unintended effects at work. However, it appears to be challenging as little research has been done to investigate the adaptivity of paper-based dietary reporting tools for promoting healthy eating in the working context. Moreover, paper-based tools, such as Doodling, could be advantageous for dietary self-reporting at work. Integrating the coloring approach into Doodling is also suggested to improve the engagement of the entire self-reporting process. The use of experience of Doodling in the working context is still left largely unexplored. Thus, understanding using acceptance of paper-based tools like Doodling among office workers is necessary for further development of dietary self-reported tools, especially in the working context.

## 3 Design of the NutriColoring toolkit

### 3.1 Key features of the design

Grounded on the users’ demands of self-reported methods identified in our prior research (Pan et al., 2021), we discovered that office workers prefer to utilize tangible supplies for a creative and playful self-reporting practice, rather than depending on mobile applications in their working contexts. Other research studies revealed that employing self-reported Doodling (Clark and Dünser, 2012; Xi et al., 2022) and integrating colors to symbolize different food categories (MyPlate | U.S. Department of Agriculture, n.d.; Aschemann-Witzel et al., 2013; Turner-McGrievy et al., 2016) are two acceptable and enjoyable approaches for health-related stimulation. As a result, we designed NutriColoring, a paper-based self-reported toolkit that integrates the Doodling and coloring approach. The design explores how Doodling and colors could be incorporated and connect working-age users with reports of daily intake by using tangible supplies (i.e., a calendar on the working desk in this study). The aim of the NutriColoring toolkit is to motivate office workers to report and reflect on personal intake patterns in daily working contexts. The NutriColoring toolkit is used as a research probe in this study with two main features.

**Figure 1:**
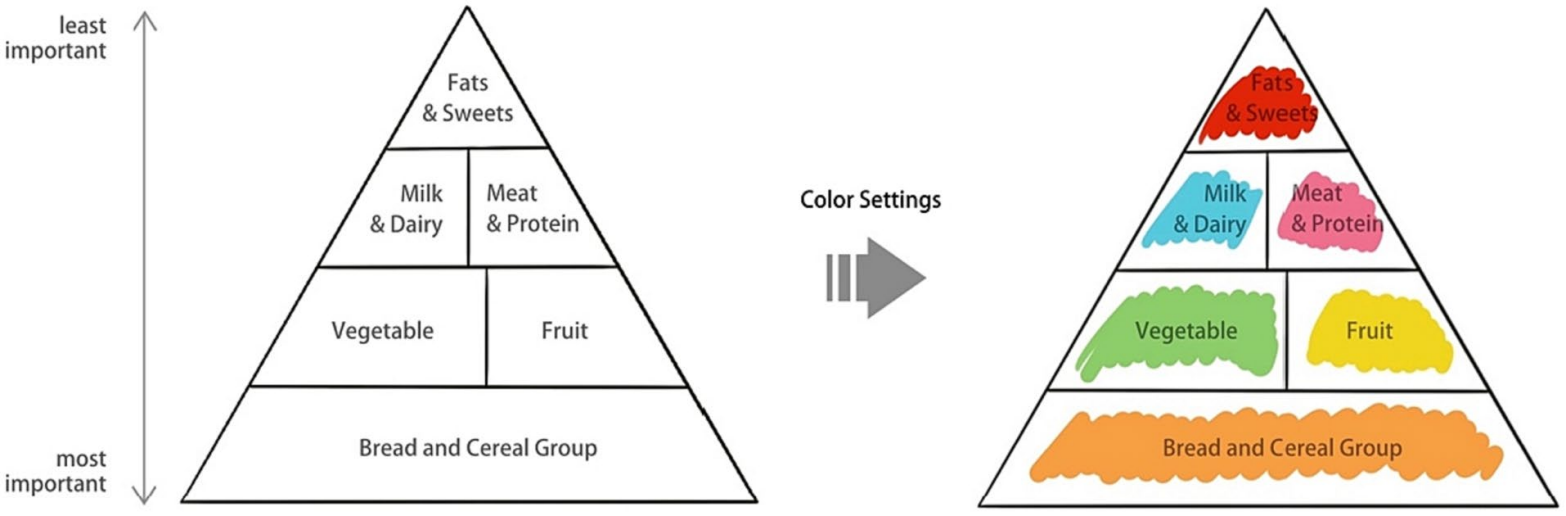
Food Pyramid and color settings.

**Figure 2:**
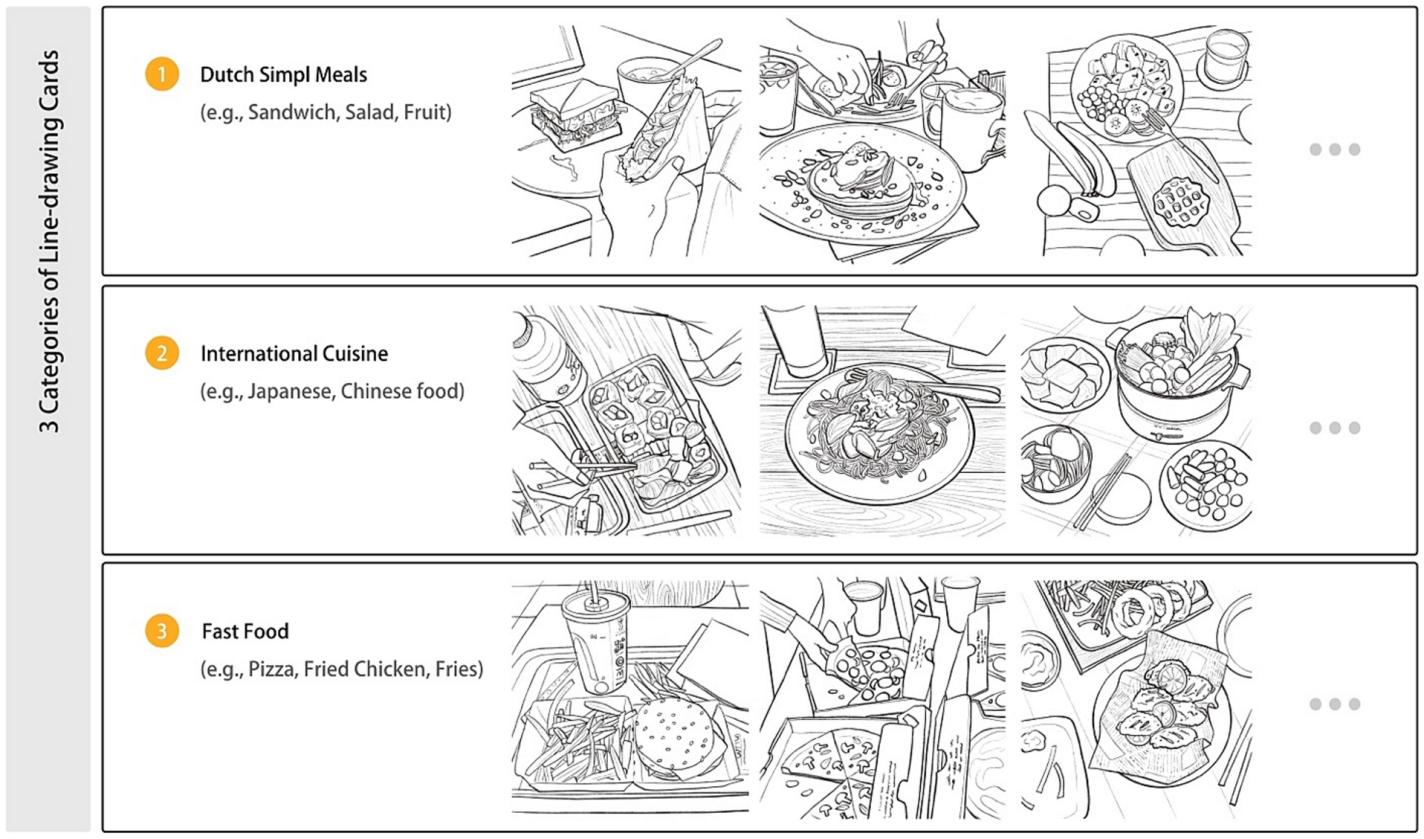
Three categories of pre-made line-drawing cards.

- *Doodling based on the Food Pyramid*. The traditional triangle-shaped Food Pyramid, initially with six food groups in the 1990s (Nestle, 1993) and revised in 2005 (U.S. Department of Health and Human Services, 2005), was later developed into MyPlate in 2011 (MyPlate | U.S. Department of Agriculture, n.d.), featuring only five food groups and excluding the unhealthy food group—fats, oils, and sweets—from the pyramid. However, there is evidence indicating that the remote working mode, which includes a shift between office and home office, has led to an increase in the consumption of convenience foods, junk foods, more frequent snacking in-between meals, and an uptake of ready-to-eat foods that are high in fat, sugars, and salt (Ammar et al., 2020; Di Renzo et al., 2020; Sidor and Rzymski, 2020). Therefore, for the design of the NutriColoring toolkit in the working context, we included the Food Pyramid (U.S. Department of Health and Human Services, 2005) as a reference. Specifically, four major shelves are included in the Food Pyramid to organize foods (as shown in Figure 1). The top shelf is the least important, while the bottom shelf is the most important. Additionally, from top to bottom shelves, the following six food groups are listed: fats, spreads, and oil; dairy; meat and alternatives; vegetables and salad; fruit; and bread and cereal food. Inspired by prior research (Burton and Baxter, 2019) that utilized colors for grouping different food categories and easy understanding of nutrition-related information, we designated six comparable colors to each of the six food categories in the NutriColoring toolkit: orange for grains, green for vegetables, yellow for fruits, blue for milk and dairy, pink for meat, and red (signifies a health risk) for fats, oils, and sweets. The coloring settings of the Food Pyramid were applied with a group of line-drawing illustrated cards and were then used for Doodling for reporting intake at work.
- *Doodling based on well-designed illustrations*. Inspired by current well-designed coloring tools (Mantzios and Giannou, 2018; Coward, 2023), one member of the research team (the first author) created a set of line-drawing cards that were used as coloring aids to make higher engagement during report intake in this study. As shown in Figure 2, we designed illustration cards in three categories for Dutch office workers. Three categories were in terms of Dutch Simple Meals (e.g., sandwiches, salads, and fruits), International Cuisine (e.g., Japanese, Chinese, and Italian food), and Fast Food (e.g., pizza, fried chicken, and French fries). These graphic line-drawing cards, which functioned like a coloring book, were used to report users’ intake by assigning colors to the specific cards based on the type and quantity of food consumed at work.

### 3.2 Design and development of the toolkits

**Figure 3:**
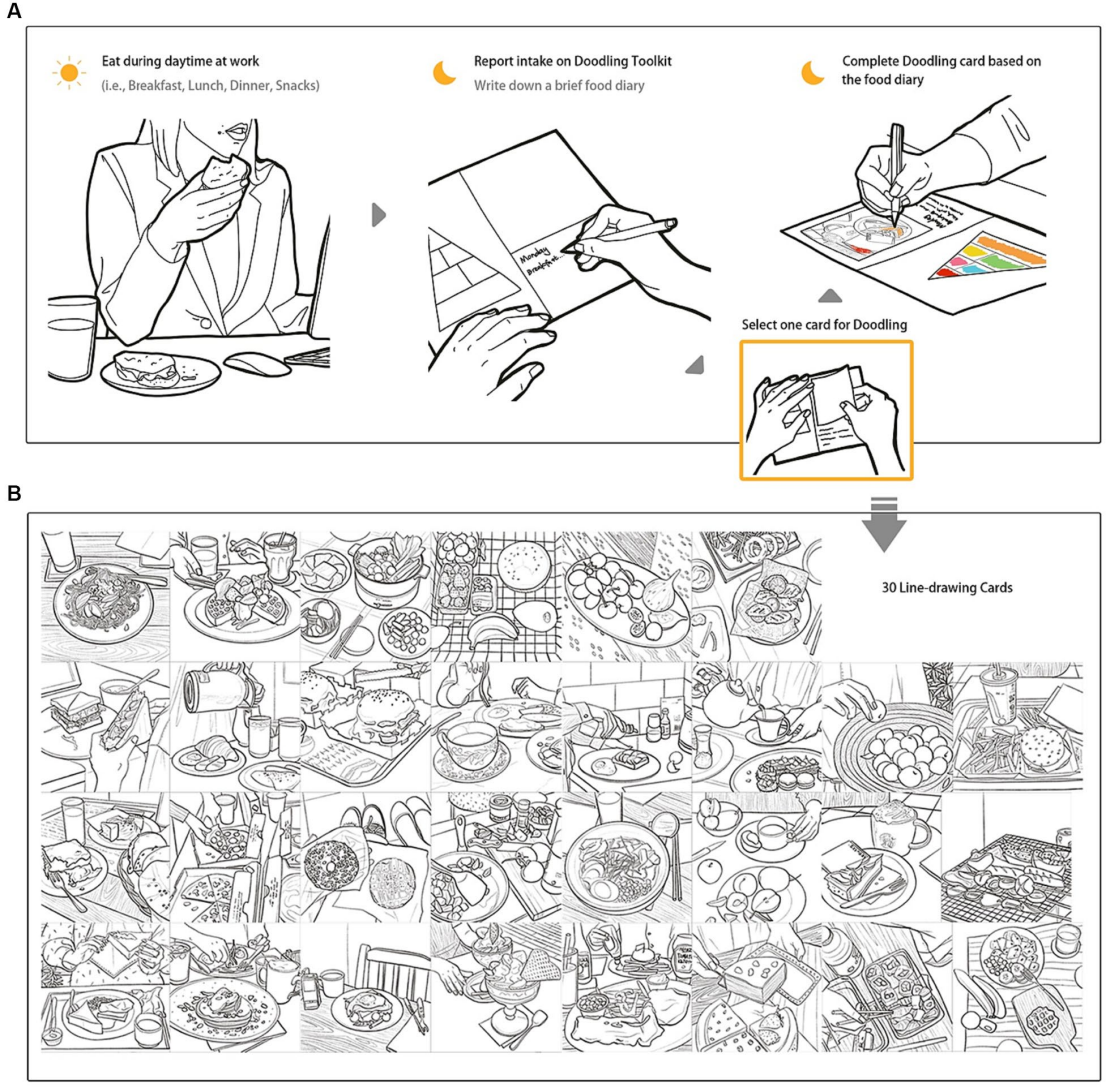
Self-reported Doodling Toolkit. **(A)** Doodling with NutriColoring toolkit; **(B)** 30 illustrated line-drawings cards for coloring the toolkit.

The design of the NutriColoring toolkit was inspired by the design guidelines of the probe toolkit (Sanders and Stappers, 2014). With the combined aims of self-reported Doodling and awareness of healthy intake at work, we identified and developed a typical using scenario for reporting activity with NutriColoring: *Coloring your daily intake as doodling*. This designed toolkit was then put into daily practice as a tangible probe and aimed to promote engaging self-reported Doodling with awareness of individual intake through displaying coloring results. Based on two key features of the design mentioned above, we developed the NutriColoring toolkit with the following content:

- *Self-reported Doodling with colors*. A tangible Doodling calendar that presents the Food Pyramid with color settings and contains seven pages for reporting intake at work (as shown in Figure 3A). A 1-week timeslot for doodling was chosen, as it aligns with office workers’ working schedules and preferences to practice weekly reporting. Besides, after asking and testing the size of each page with participants, 20 cm x 15 cm was used as the size of the toolkit as it fits on a working desk without taking up too much space.
- *30 line-drawing cards with various meal types*. Thirty line-drawing cards have been designed and developed for this study (see Figure 3B). Each day, users could pick one of 30 cards that most accurately reflects their food consumption and then draw it using six colors categorized by the Food Pyramid. The sequence of the colored cards on the Doodling frame may be arranged differently for each user as various users have unique eating patterns.

**Figure 4:**
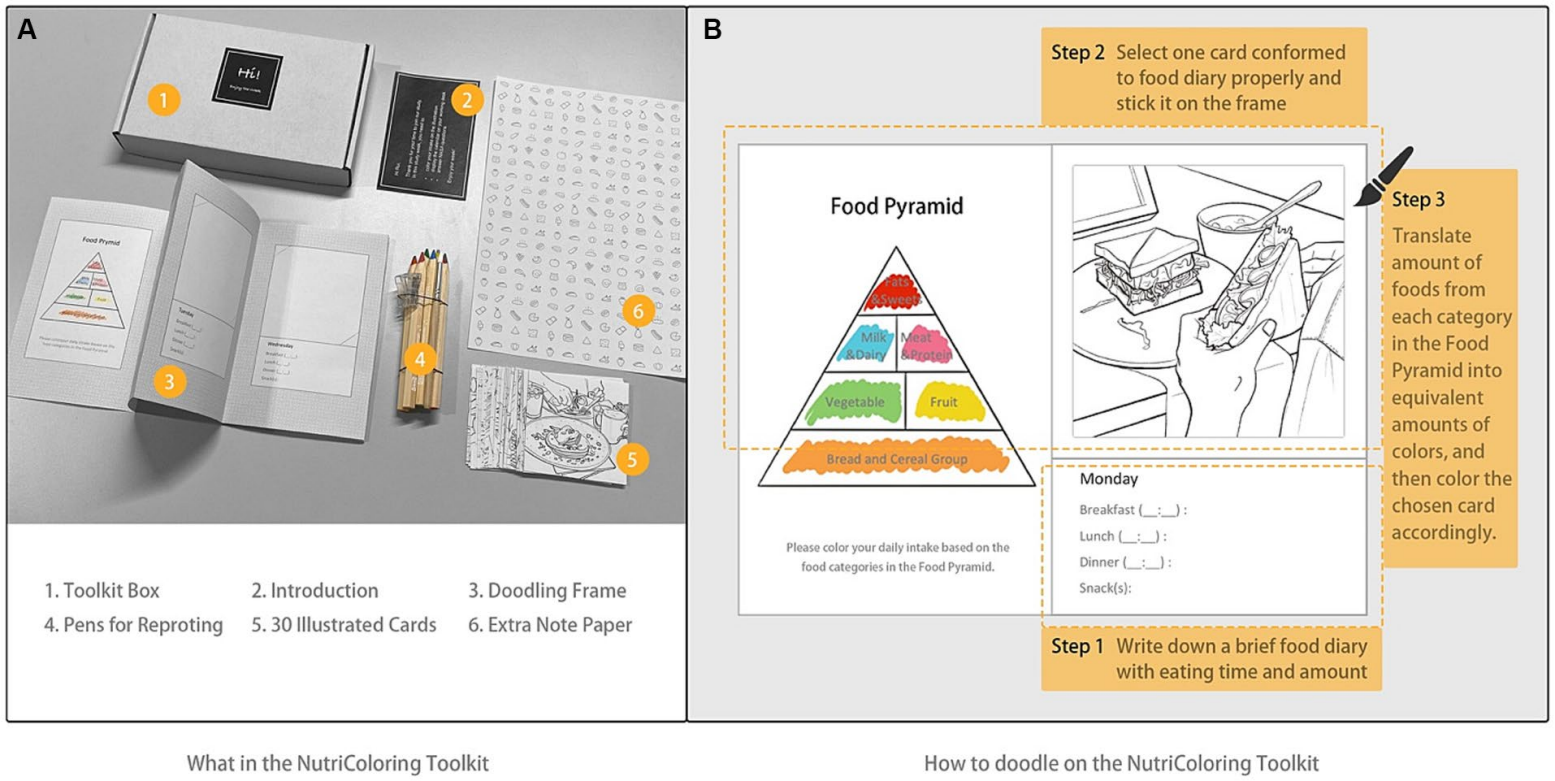
Contents of NutriColoring probe toolkits. **(A)** Items in the NutriColoring Toolkit; **(B)** How to doodle on the NutriColoring Frame.

Eventually, the NutriColoring toolkit (Figure 4) has been designed as a box with a 1-week Doodling frame, 30 line-drawing cards, six colored pens (orange, green, yellow, red, blue, and pink), and an introduction to how to use the toolkit. Upon receiving the toolkit, users have the flexibility to determine when, how many times per day, and where to use it based on their work routines, schedules, and personal preferences. During the usage of NutriColoring, the user should first write down a brief food diary (i.e., eating time, intake amount of meals, and/or snacks) on the doodling frame each day; select one line-drawing card that conforms to their daily intake properly; color the card with pens based on the eating amount of each of the food categories; stick the card onto the doodling frame; and display the frame on the working desk during the entire study week.

**Figure 5:**
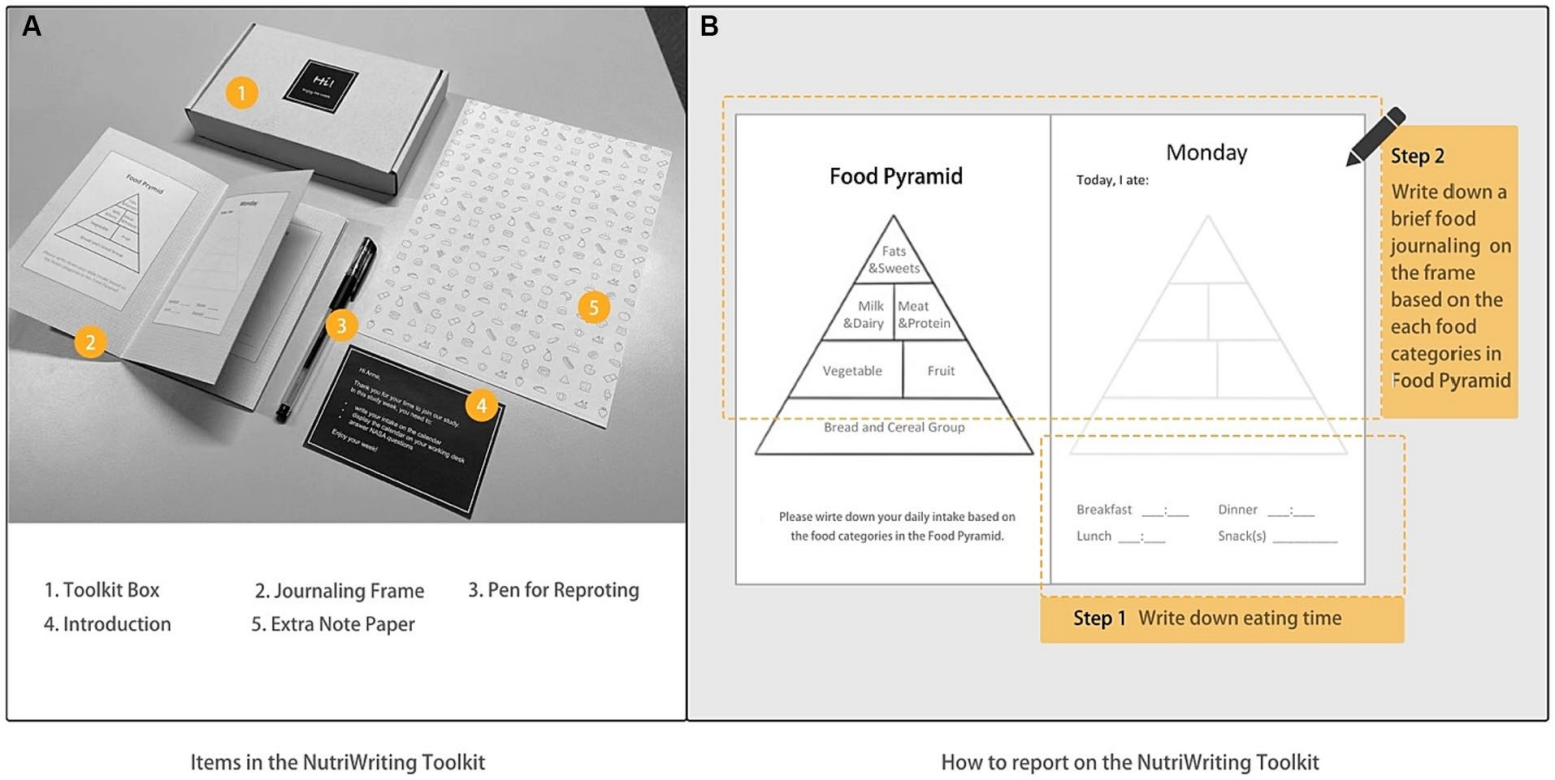
Contents of NutriWriting probe toolkits. **(A)** Items in the NutriWriting Toolkit; **(B)** How to report on the NutriWriting Frame.

To investigate the benefits and to determine the advantages of the NutriColoring toolkit in the working context, we compared it to a writing approach by removing the color settings of the NutriColoring toolkit. The NutriColoring meant the participants were to draw the doodles on a paper-based probe to illustrate the food intake, while the prompt “writing” meant they were to write out the text-based food intake every eating time. This uncolored toolkit in the form of traditional food journaling way was named NutriWriting in this study. As seen in Figure 5, NutriWriting was created with a page introducing the Food Pyramid and seven pages for reporting one-worth weeks of intake while working. The NutriWriting toolkit has 1-week Journaling with a pen and an introduction inside the box. While using NutriWriting, the user needs to write down the specific eating time and their intake according to the Food Pyramid and then display the Journaling frame on their working desks.

## 4 The study and method

In response to the research questions, the objectives of the user study were to investigate (1) the effectiveness of NutriColoring in facilitating self-reporting of daily intake in the working context; (2) the role of NutriColoring in stimulating awareness and self-reflection on daily intake. We used a within-subject design, with participants reporting daily intake at work with two toolkits (NutriColoring vs. NutriWriting) mentioned above. We compared two approaches relating to the user experience from both quantitative and qualitative aspects. Our primary hypothesis is as follows:

*H01*: The Doodling via coloring approach (NutriColoring) will be more effective in reporting daily intake at work than text-based food Journaling (NutriWriting).

The participant characteristics, the study procedure, and data collection and analysis are all included in the following section.

### 4.1 Participant

We recruited participants by spreading information via word of mouth, using a snowball sampling approach. Firstly, we asked people we know who suit our recruitment criteria for the target groups. We then asked them to pass the information via Facebook, Twitter, or other social media like WhatsApp to their social contacts. During the recruitment, we screened participating candidates based on the following criteria: (1) knowledge workers who are engaged in a job that requires desk/computer work for min. 24 h/week in the office or work from home (due to COVID-19); (2) do not follow any dietary treatments; (3) are interested in coloring approach but do not have a background in the creative disciplines; and (4) are not color-blind people. They were fully informed of the study procedure before the study weeks and were given the opportunity to withdraw at any time. In total, 20 participants were recruited, and two of them were excluded as they did not have any experience with the coloring approach and did not work in a fixed place. Finally, 18 participants (gender: 8 males and 10 females, age: *M* = 29.7, SD = 4.91, Min = 25, Max = 46) completed the entire study. Almost all participants gained a certain educational level (three with bachelor’s degrees, 11 with master’s degrees, and four with PhDs) and have worked at least half a year (*M* = 4.64 years, SD = 5.59). Due to the COVID-19 pandemic, they chose hybrid working contexts (work from home and work in an office). Their characteristics are summarized in Table 1. We labeled the 18 participating dyads as P01, P02 … P18 in this study.

**Table 1:** Characteristics of 18 participants and their eating goals.

	Gender	Age	Education level	Working years	Working hours/day	Eating goal at work
P1	Female	29	Master	2.5	6–8	Healthy eating of non-processed food
P2	Male	32	Master	4.5	6	Gain more protein
P3	Male	29	Master	3	6–8	Gain more weight and protein
P4	Female	26	Master	1	6–8	Diet following 8–16 eating method
P5	Female	26	Master	1.5	6–8	Eat more vegetables and fruits
P6	Female	26	Master	1.5	6–8	Eat healthier and control weight
P7	Male	31	PhD	4.5	8	No heavy lunch
P8	Female	27	Master	0.5	6–8	A balance of different nutrition
P9	Male	25	Master	1	14	Have enough energy to do all my tasks
P10	Male	27	Master	1	8	Eat less processed but nutritional food
P11	Female	46	Bachelor	20	8	Nothing special
P12	Female	29	Bachelor	5	9	Eat nice meals as I like
P13	Male	30	Master	7	8	Keep meat consumption low
P14	Female	31	Master	2	8	Varied-nutrient diet
P15	Female	29	PhD	3	6–8	Eating healthy with more food choices
P16	Male	32	Master	7	8	Nothing special
P17	Male	35	Bachelor	1	8	Low carb diet
P18	Female	25	Master	0.5	8	Eat less fat and sweet

### 4.2 Study procedure

**Figure 6:**
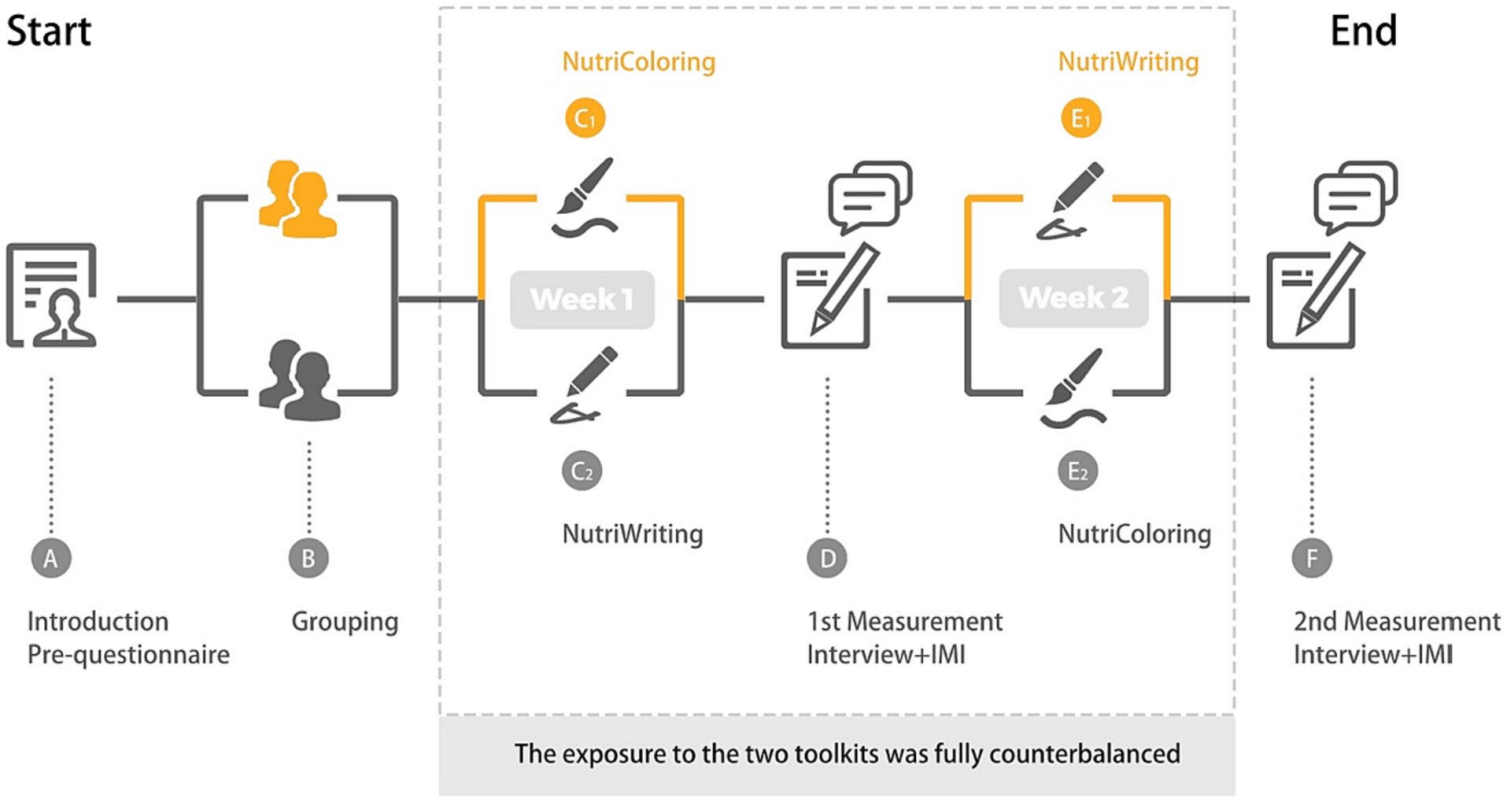
Visualization of the study procedure.

The cultural probe study was approved by the Ethical Review Board of the Eindhoven University of Technology (reference: ERB2021ID97). We conducted this study between February and March 2022 in the Netherlands. As shown in Figure 6, the study initially had an introduction session to explain the study procedure without discussing the research hypothesis. Each participant was asked to sign a consent form and to complete a pre-questionnaire regarding their demographic information, eating goals, and working status. Then, they were randomly given one of NutriColoring and NutriWriting for the first study week, and another toolkit for the second study week, with a washout week between the 2 study weeks. The exposure to the two probing packages was fully counterbalanced. Participants were encouraged to maintain their usual eating practices, whether it involved eating in the office canteen or bringing their own self-made food. They were given the flexibility to choose when, how often, and where they used the toolkit, aligning with their individual work routines. As part of the research process, participants were required to describe how many minutes they spent using each toolkit and complete the NASA-TLX index (Hart, 2006) daily for the workloads. At the end of each study week, we asked participants to take an Intrinsic Motivation Inventory (IMI) (McAuley et al., 1989) to measure the using experiences of two reporting approaches (NutriColoring vs. NutriWriting). Afterward, we conducted an in-depth interview session with each participant individually.

### 4.3 Measurements

As shown in Table 2, we collected both quantitative and qualitative data for two reporting approaches. First, the evaluation of the user experience mainly focuses on mental workload and intrinsic motivation (Deci and Ryan, 2013). In this study, we used NASA-TLX (Hart, 2006), a tool designed for assessing subjective mental workload, to measure the mental workload experienced by participants while using the NutriColoring and NutriWriting toolkits. We maintained two subscales of NASA-TLX related to our study purpose: mental demand and frustration. NASA-TLX aimed to indicate how burdensome the participants felt the reporting approaches during their working hours, which might negatively influence the engagement in reporting intake. All subscales were rated from 1 to 21; low ratings represent a lower workload. Moreover, the participant’s intrinsic motivation to carry out the intake reporting was measured by IMI (McAuley et al., 1989). IMI contains 45 items across seven subscales, which asses self-desire for a specific activity. We selected five subscales due to their high relevance to our study purpose, including interest/enjoyment, perceived competence, pressure/tension, effort/importance, and value/usefulness. Each subscale should be scored from 1 (not at all true) to 7 (very true).

**Table 2:** Data collected from the study.

Measures	Week 1							Week 2						
	Day 1	Day 2	Day 3	Day 4	Day 5	Day 6	Day 7	Day 1	Day 2	Day 3	Day 4	Day 5	Day 6	Day 7
NASA-TLX	●	●	●	●	●	●	●	●	●	●	●	●	●	●
IMI							●							●
Follow-up interview							●							●

After each study week, a semi-structured interview was conducted for approximately 30 min per participant to collect qualitative data regarding user experience and opinions on NutriColoring and NutriWriting. The interviews followed a pre-set protocol guided by a qualitative interview technique (Blackstone, 2018) and included open-ended questions about intake reporting and the influence of self-awareness. The questions were set based on TAM-Usefulness (Holden and Karsh, 2010) and Usability Risk Level Evaluation (Jin and Ji, 2010). We keep the questions that suit our research purposes and aims. “What do you like and dislike about reporting your intake with the toolkit last study week?,” “Does the toolkit help you to be aware of your intake quality?,” and “What factors influenced your user experience with the toolkit last week?” To elaborate on participants’ experience with NutriColoring and NutriWriting at work, we then asked them questions such as “How would you rate your eating practice in the past week?” and “Could you please share the stories about your experience related to the reporting approach in the past week?” We also asked participants to explain more interesting statements that emerged during the interview. All interview sessions were audio-taped and transcribed later for qualitative analysis.

### 4.4 Data analysis

#### 4.4.1 Quantitative analysis

The NASA-TLX IMI questionnaire data were analyzed via SPSS software (SPSS, IBM Version 26; SPSS, Inc., Chicago, IL). First, the quantitative data were processed with descriptive statistics, in which the distribution of the NASA-TLX and IMI data was checked through Shapiro–Wilk tests. For data with normality, we conducted paired-samples *t*-tests with the two self-reporting toolkits (NutriColoring vs. NutriWriting) as a factor. For the data that were not normally distributed, we conducted a Wilcoxon signed-rank test to measure the difference between the two approaches. The effects of the two toolkits and the day of the study week (from Monday to Sunday) on the workload were also evaluated using a two-way ANOVA. The main objectives of our quantitative analyses were to (1) test the task load of both NutriColoring reporting week and NutriWriting reporting week; and (2) test the intrinsic motivation of the two approaches in the working context.

#### 4.4.2 Qualitative analysis

The results of the interviews were collected and analyzed via MAXQDA software. The thematic analysis (Braun and Clarke, 2006) following inductive coding (Thomas, 2006) was used for data analysis with the following steps: First, the segmentation of the transcripts was transformed into quote statements and labeled. Then, the labeled statements were measured using inductive coding to identify recurring clusters with emergent themes (Thomas, 2006). Additionally, the analysis of statements was counted to indicate the relevance to our quantitative data. Next, all identified themes and clusters were reviewed, discussed, and revised through several iterations with most members of the research team (the first, second, and third authors) to validate the qualitative analysis. The purpose of qualitative analysis is to understand the user experience of the self-reported approach with coloring way compared to the writing approach and then develop further design opportunities for dietary reporting tools with the Doodling probe (the NutriColoring toolkit).

## 5 Results

### 5.1 Quantitative findings

#### 5.1.1 Workload

**Figure 7:**
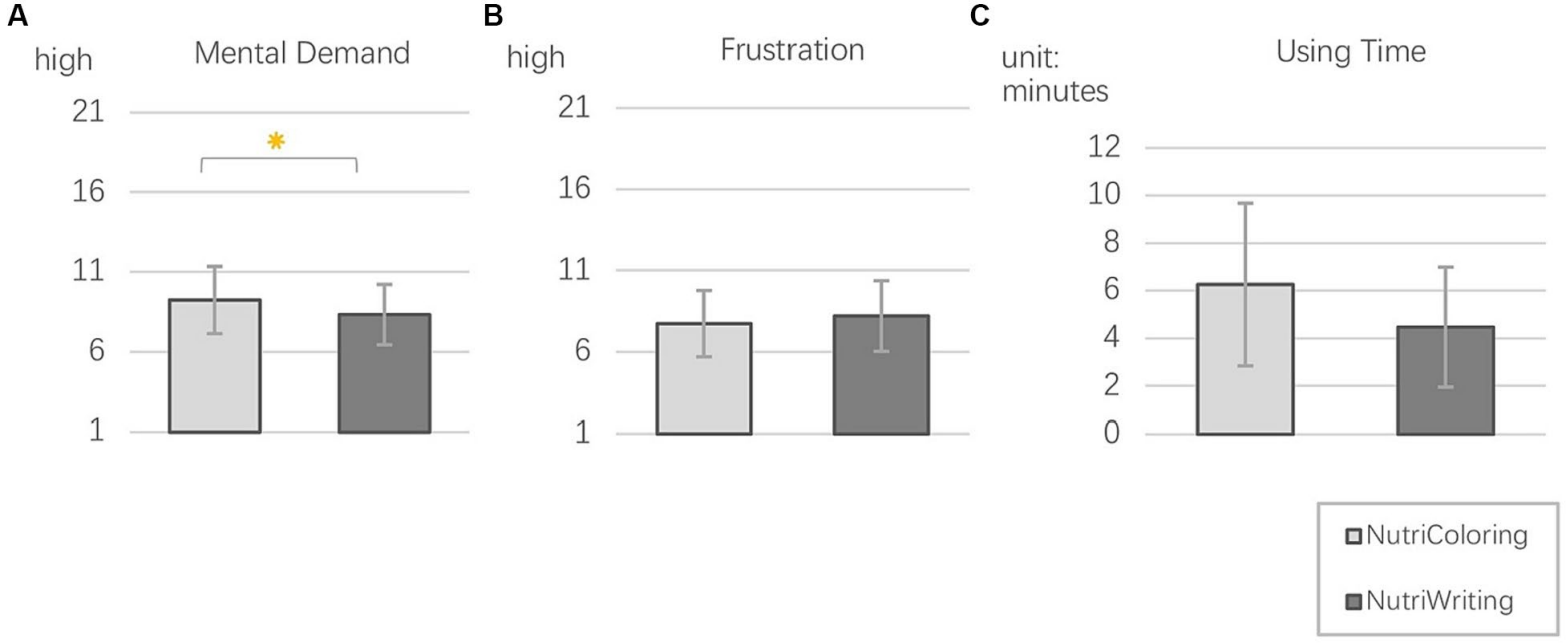
Mean and SD of NASA-TLX sub-dimensions: **(A)** Mental demand; **(B)** Frustration; **(C)** Using time with two toolkits.

The workload was measured via NASA-TXL with two subscales: *mental demand* and *frustration*. We also asked about using time with each toolkit during the study weeks. Regarding the *Mental demand* (see in Figure 7A), reporting with the NutriColoring (*M* = 9.23, SE = 0.19) was reported to require a higher cognitive load than reporting with NutriWriting (*M* = 8.32, SE = 0.18). A Wilcoxon signed-rank test indicated that there was a significant difference between the two approaches, *z* = −2.04, *p* = 0.042, with relatively large effect size, *r* = 0.48. Regarding the *Frustration* (shown in Figure 7B), we found that the using frustration with NutriColoring (*M* = 7.73, SE = 0.18) was lower than with NutriWriting (*M* = 8.20, SE = 0.19). According to the Wilcoxon signed-rank test, the differences were not significant, *p* = 0.289. Regarding the time consumed for self-reporting approaches (as shown in Figure 7C), participants used less time reporting intake with NutriWriting (*M* = 4.47 min, SE = 0.21, Max = 25 min, Min = 3 min) than with NutriColoring (*M* = 6.26 min, SE = 0.29, Max = 16 min, Min = 3 min). In addition, the Kruskal–Wallis test showed that there is a significant difference in using time between the NutriColoring toolkit and the NutriWriting toolkit (*p* < 0.001).

**Figure 8:**
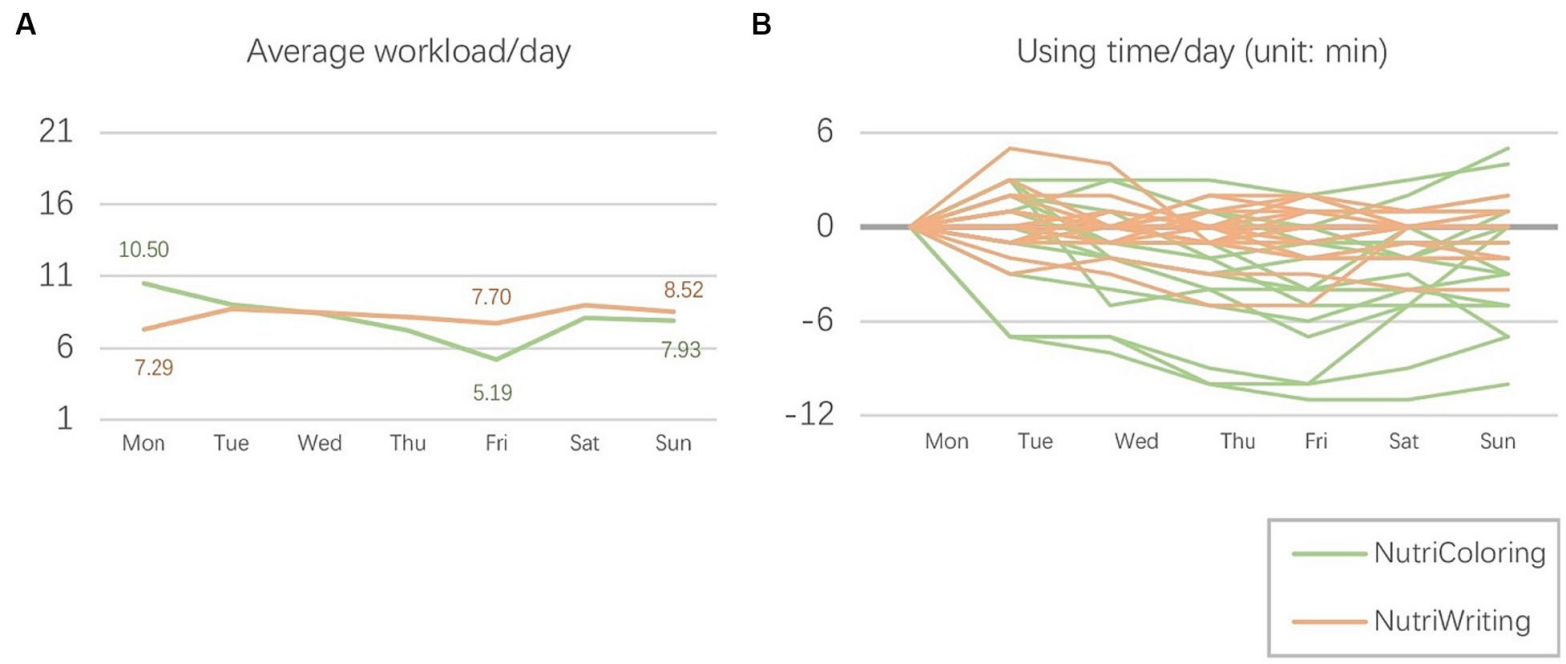
**(A)** Average workload per day and **(B)** The using time per day of two toolkits.

Besides, comparing the average data over the entire study, we also analyze the NASA-TLX scores and task completion durations on a daily basis to understand the changes in workload throughout the study. The two toolkits’ separate average daily workloads over the period of a week were calculated for the comparative analysis, and each is shown in a line graph in Figure 8A. Overall, we found that the mean workload increased while using the NutriColoring toolkit, but the situation was the total opposite when using the NutriWriting toolkit. It was interesting that the workload for NutriColoring began to fall below that for NutriWriting on the third day of the study procedure. Specifically, starting from a relatively higher level of mental demand and reporting frustration (M = 10.50), the figure of NutriColoring then decreased slightly. The average workload of the coloring approach dropped to its lowest point on Friday (*M* = 5.19) but quickly rose to a high level throughout the weekend, ending with a mean of 7.93. In contrast, among the 18 participants, the average workload for the writing approach started lower (*M* = 7.29) but then increased slightly. Even while it also hit a low point (*M* = 7.70) on Friday, the scores rose over the weekend, concluding with a mean of 8.52. To determine the impact of toolkits (NutriColoring vs. NutriWriting) and study days (from Monday through Sunday) on workload, a two-way ANOVA was conducted. There was no significant main effect of the two self-reporting approaches on workload, *F* = 0.18, *p* = 0.67, and no significant difference in study days, *F* = 0.14, *p* = 0.99.

On the other hand, we also measured how much time each participant spent each day using two toolkits during the study process. The participant’s usage time on the first day of every study week is taken as a baseline. A decrease in the time spent (expressed as negative time costs in the figure) indicates an improved user experience and reduced learning costs. Figure 8B illustrates the changes in the time spent using both the coloring approach in a 1-week-long period and the writing food diary approach in another week. Compared to the first-day baseline, using time with the NutriColoring toolkit (*M* = −2.02, SD = 2.76, Min = −8.14, Max = 2.29) showed a gradual decline, while the time with the NutriWriting toolkit (*M* = −0.12, SD = 1.04, Min = −2.71, Max = 1.14) fluctuated even a little using time reached a peak on the second day. Furthermore, the use time of both toolkits presented a slight climb during the weekend, especially the using duration with the NutriColoring toolkit was increased during weekends.

In summary, self-reporting behaviors with our toolkits scored relatively low in the NASA-TLX workload survey. Reporting intake with the NutriColoring toolkit during working hours seemed to require more mental demand but less frustration from participants compared to the NutriWriting toolkit. These results suggest that the using of NutriColoring may play a positive role in enhancing user experience with intake assessment in the working context, which might be used to sustain users’ engagement in the long term.

#### 5.1.2 Intrinsic motivation

**Figure 9:**
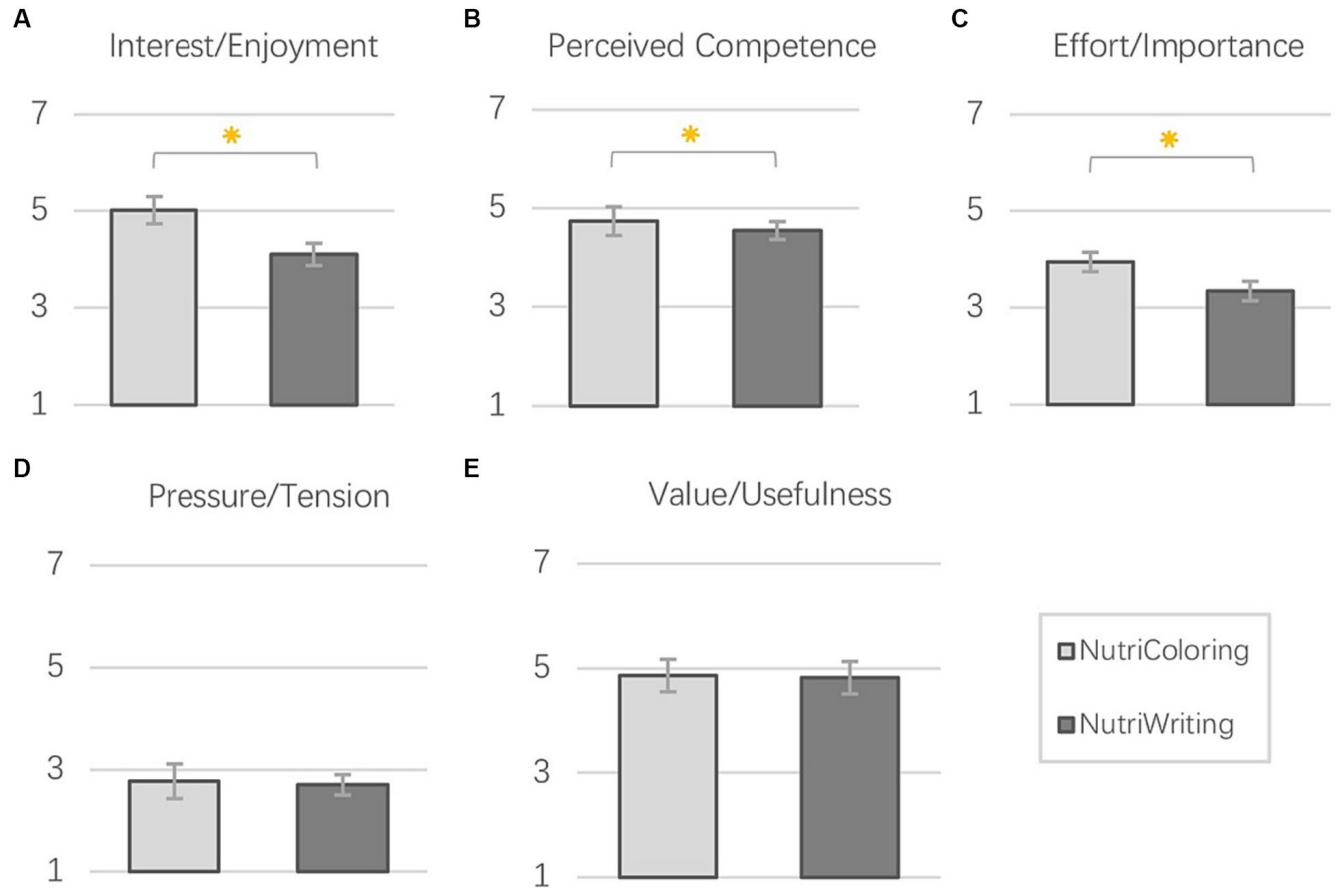
Mean and SE of IMI subscales: **(A)** Interest/Enjoyment; **(B)** Perceived competence; **(C)** Effort/Importance; **(D)** Pressure/Tension; **(E)** Value/Usefulness.

Figure 9 shows the results of the IMI questionnaire. Overall, we found that participants were positively motivated to report daily intake during working hours, with reasonably high scores on the subscales of *interest/enjoyment, perceived competence, and value/ usefulness*. Additionally, ratings for these two approaches (coloring vs. writing) were moderate for the subscale of *effort/importance* and low for the subscale of *pressure/tension*. The quantitative analysis with paired-samples *t*-tests showed significant differences in *Interest/enjoyment*, *Perceived competence*, and *Effort/importance* between the two reporting approaches at work.

##### 5.1.2.1 Interest/enjoyment

Figure 9A shows a significant difference in enjoying the reporting process with the Doodling via Coloring approach and the traditional text-based food Journaling (*t* = 3.491; *p* = 0.003). The *Interest/enjoyment* was rated significantly higher for the NutriColoring (*M* = 5.01; SE = 0.28) than for NutriWriting (*M* = 4.10; SE = 0.23).

##### 5.1.2.2 Perceived competence

As shown in Figure 9B, there were significant differences between the two approaches of reporting intake during working hours (*t* = 2.884; *p* = 0.010). Participants felt the perceived competence with using the NutriColoring toolkit (*M* = 5.07; SE = 0.20) was significantly stronger than with the NutriWriting toolkit (*M* = 4.56; SE = 0.19) while they worked.

##### 5.1.2.3 Effort/importance

In the *Effort/importance* subscale (Figure 9C), the rates of all participants were also significantly different for the two reporting approaches during working hours, *t* = 2.314, *p* = 0.033. The intake reporting activity was considered significantly more important with the NutriColoring (*M* = 3.87; SE = 0.21) than with NutriWriting (*M* = 3.31; SE = 0.22).

##### 5.1.2.4 Pressure/tension and value/usefulness

On both two subscales, NutriColoring was rated higher than NutriWriting. However, regarding the perceived tension of the intake reporting at work (shown in Figure 9D), there was no significant difference between the NutriColoring (*M* = 2.77; SE = 0.34) and NutriWriting (*M* = 2.70; SE = 0.20), *p* = 0.924. Regarding the perceived usefulness of the intake reporting (as shown in Figure 9E), the value of NutriColoring (*M* = 4.86; SE = 0.31) is slightly higher than that of NutriWriting (*M* = 4.82; SE = 0.29). There also was no significant difference between the Doodling and the writing Journaling, *p* = 0.660.

### 5.2 Interview results

#### 5.2.1 NutriColoring

According to the follow-up interviews, all participants preferred using the NutriColoring toolkit for reporting daily intake in the working context. Their reasons for their choice could be summarized as follows.

*First*, the interview results indicated that most participants expressed a positive attitude toward the playful user experience with the NutriColoring toolkit. They stated that they could see potential benefits of the Doodling with Coloring approach for self-reporting during their working hours and even for long-term use. For example, P4 mentioned “*It relaxed my mind from work, and I looked forward to using the toolkit every working day.*” Ten participants described the use of the NutriColoring toolkit as “*enjoyable*” and “*interesting,*” and six participants described it as “*exciting*.”

*Second*, the responses indicated that the 30 illustrated cards were efficient in motivating a flexible and creative using process and helped protect privacy at work. For instance, P3 explained: “*It strongly encouraged me to enjoy coloring when the illustrated card I chose perfectly corresponds to my daily intake. I also like to select cards in advance and plan my meals with healthy food choices for the following day(s).*” Other participants stated: “*It is simple to remember the food groups and colors. After that, I felt more freedom and less pressure in self-desired drawing ways. For example, a banana in green, or orange with meat textures* (P6).” Participants also mentioned that the flexibility of creation helped to hide their specific intake and protect their privacy in the working context. P5 stated “*Others cannot understand my cards since they were casual creations and only I know the content in detail*.”

**Figure 10:**
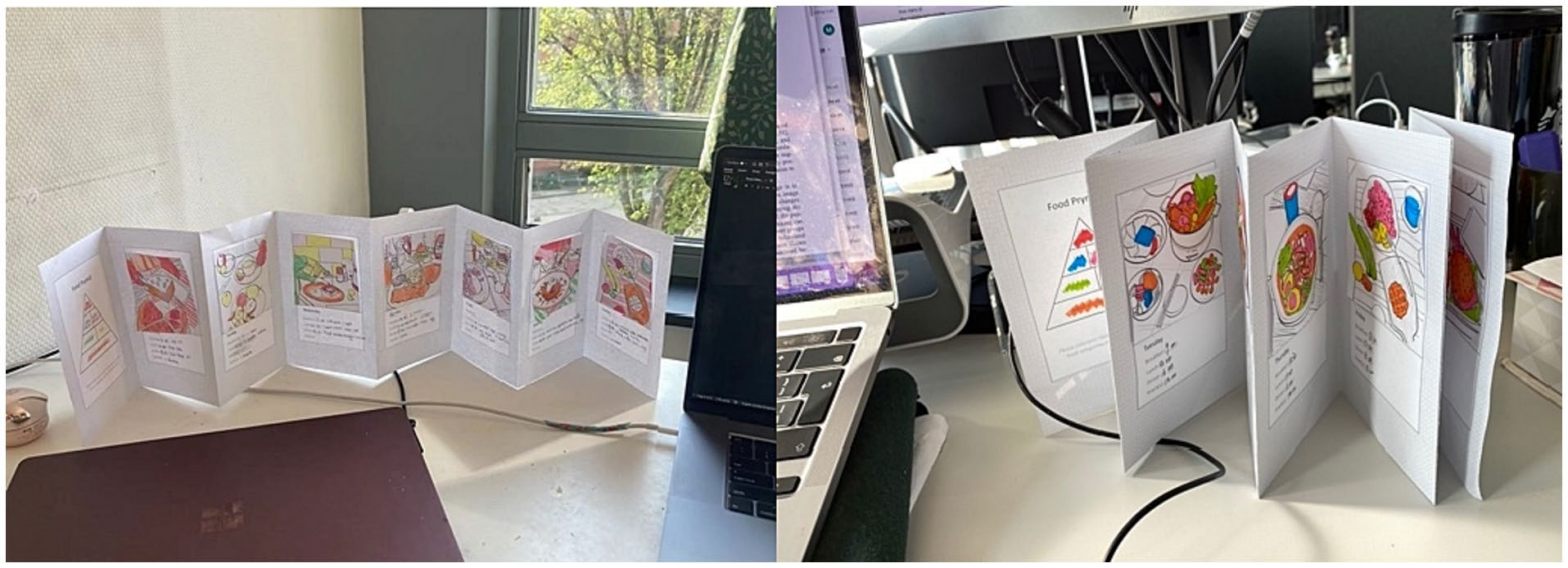
Examples of displaying results of NutriColoring toolkit on participants’ working desks.

*Third*, the NutriColoring toolkit was seen as a self-reflection enhancer by the majority of participants. Through collecting images of colored toolkits taken by participants, we noticed that most participants preferred to display colored results in a prominent location on their working desks (as shown in Figure 10). For instance, P2 stated “*It gave me a sense of personal achievements while I put the toolkit on my desk as a piece of art.*” Participants explained that the display of their reporting data could “*provide a clear overview of intake history*,” “*compare personal intake with food groups in Food pyramid reference*,” “*directly recognize the missing or overeating of a certain food group(s)*,” and “*trigger to balance the intake*.” P3 also mentioned that: “*Compared to texts, colors are well-visualized feedback, which encouraged me to improve food diversity and keep eating as good as/better than previous days*.” These findings are in line with quantitative results that the NutriColoring toolkit is an interesting and valuable approach for reporting daily intake in the working context.

**Figure 11:**
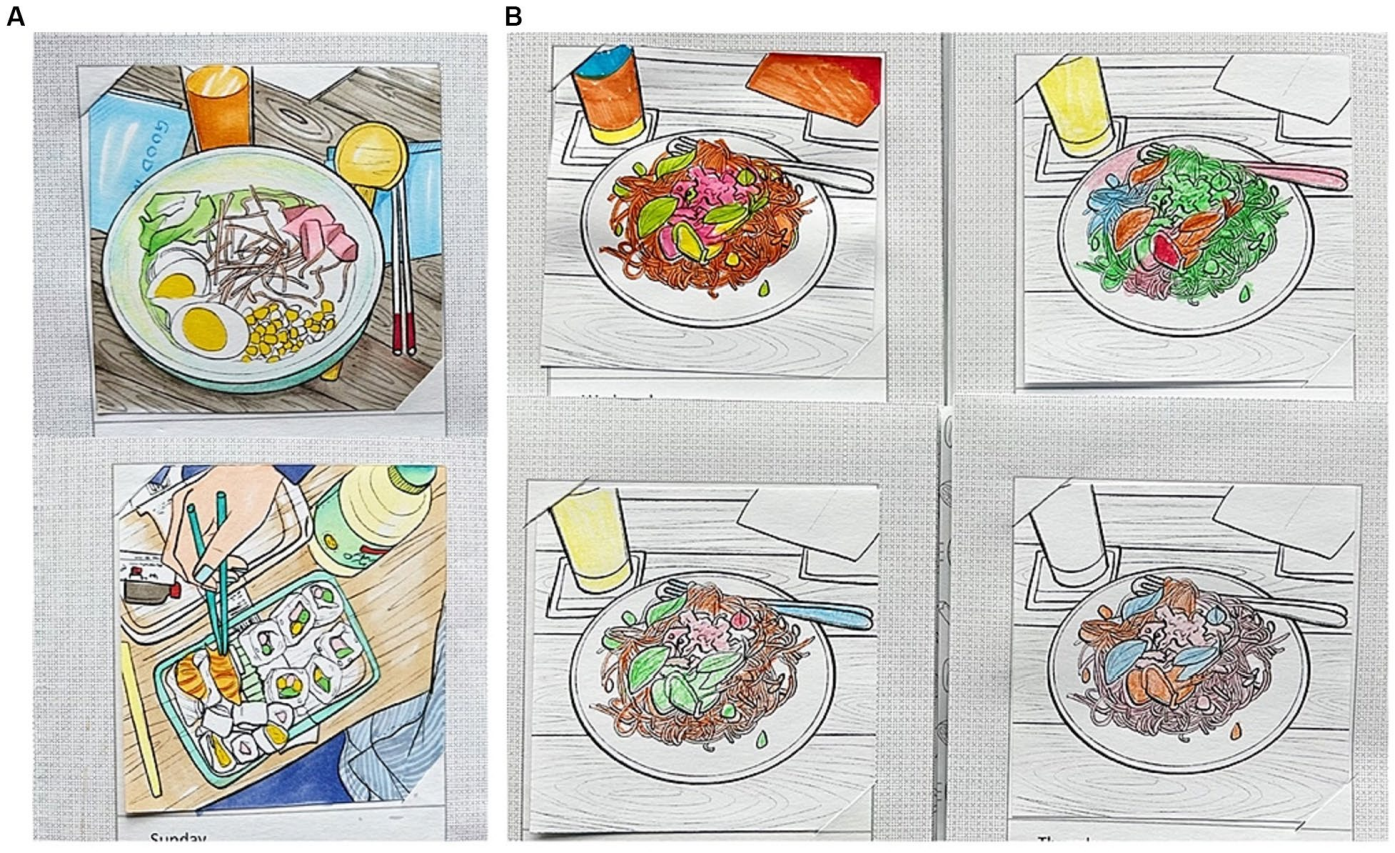
Coloring results regarding **(A)** full-size coloring way and **(B)** various eating choices among different participants with the same illustrated card.

*Additionally*, we observed that participants used the NutriColoring toolkit to color their doodles in various ways. In particular, some participants preferred coloring the entire card (as shown in Figure 11A), while others (as shown in Figure 11B) only colored food-related contents without drawing backgrounds or non-food items (such as dining table, tissues. The doodling results were strongly influenced by each participant’s eating habits and food choices. The distribution of color proportions on the same card drawn by different participants can be compared to reveal how each participant’s nutritional structure differs. For example, as shown in Figure 11B, some participants consumed more vegetables (in green), some ate more meat (in pink), and some preferred grain (in orange). Besides, 8 of the 18 participants discovered that, in contrast to NutriWriting’s text-based method, colors might visually prompt participants to adjust their food consumption by presenting varied intake amounts for each food category as well as helping individuals spot missing food groups. *However*, two participants stated different attitudes toward NutriColoring. For instance, they demonstrated that “*Coloring approach requires investing a big effort and time to get awareness of daily intake* (P1)” and “*It was playful but was a lot for me. I would prefer less stress during the reporting process* (P15).”

#### 5.2.2 NutriWriting

Only two participants selected NutriWriting as their preferred self-reporting way during working hours. They claimed that adopting the NutriWriting toolkit made the reporting process simpler to understand, easier to follow, and less using effort, all of which allowed them to maintain their attention on their current tasks. For instance, one participant mentioned, *“……writing was the easier way for me to follow without overthinking, and it was convenient to complete unfinished reporting with writing*.” This is in line with our quantitative findings that NutriWriting needs shorter time and lower efforts for reporting intake during working hours. On the other hand, some participants thought that NutriWriting lacked visualized results about their intake. For instance, as some participants stated, “*……I gained no valuable information if I only took a glance at the calendar without carefully reading,” “Compared to the coloring calendar, the text-based calendar was not helpful to raise my awareness of intake during my working hours.”* Besides, five of eighteen participants stated that they did concern about their privacy when they displayed the NutriWriting toolkit on their working desks during the research process because everyone passed by could read and know their data. Moreover, thirteen participants would not like to keep using the NutriWriting toolkit at work for the long term. They predicted the long-term using experience with NutriWriting would be “*repeated efforts,”* “*boring,*” and “*limited effect.*”

#### 5.2.3 Other findings

The qualitative analyses also showed that the NutriColoring toolkit as well as the NutriWriting toolkit can promote reporting intake during working hours in an easy and simple way. The interview responses suggested that the Food Pyramid reference enables a new form of understanding food categories and helps report intake efficiently but needs to provide the possibility to encourage participants to achieve personal eating goals. Most participants also experienced a hybrid working context during the COVID-19 pandemic, where emerged a need to design portable products for reporting intake. We elaborated on these findings below, highlighting three other aspects.

##### 5.2.3.1 Quantify intake in a simple way

Although NutriColoring and NutriWriting took the Food Pyramid as a reference, most participants stated that it was difficult to quantitatively compare their intake day by day. For instance, P5 mentioned, “*I only draw illustrated food on the card instead of coloring the entire card, because I want to tell the consumptions of each food group by changing the size and area of coloring.*” P2 explained that “*I can tell the amount of intake by seeing the word size and length on the NutriWriting toolkit, but I still look forward to a guideline to tell me whether I eat enough and healthy or not.*” Some other participants suggested that “*It would be beneficial if my the-day-before intake could be my reference, then I can learn if I behave in a better and healthier tendency or in opposite.*” Besides, colored pens in the toolkit were considered as a quantitative tool to promote understanding and decision about intake amount. For example, P4 and P5 stated, “*I thought red (represents fat) is unhealthy, so I put red pen outside the toolkit box and tried to avoid using it*.” P10 mentioned that “…*I always lined up the pens from most to least according to proportions of each food group that day. After that, I just started drawing on the cards, which helped me to realize how much I eat.*” Some participants also suggested that it would be easier to quantify intake using stickers or Lego bricks of the same size but in different colors to report intake at work.

##### 5.2.3.2 Eating goal and behavior change

In the pre-questionnaire, we asked every participant to mention their personal eating goal and most participants reported a good result in achieving their goals. Instead of finding an eating goal from a scientific institute and dietician, participants showed interest in setting eating goals according to their actual needs and status. For instance, some participants “*plan to eat more vegetables and fruits at work*,” some “*try to eat more types of food in one food category*,” and some others thought “*less fat and sweet, low carbine could help to build up a healthy physical status*.” The interview results surprised us that almost all participants were aware of their goals and tried to achieve them during the study process, even though this task was not required for this study. Furthermore, some participants also started to change their eating behaviors by setting doable small steps and challenges. For example, P15 pushed herself to eat two times more fruits than yesterday, and P2 challenged himself to keep regular eating time on a super busy workday.

##### 5.2.3.3 Hands-on coloring tasks within a hybrid working context

On the one hand, tangible toolkits also brought hands-on activity in the working context. Specifically, most participants preferred using pens to report intake, rather than with mobile applications. They explained that this hand-made approach could help them “*gain an excuse to relax from heavy working schedules,*” “*improve retention of intake information,*” and “*learn their own eating patterns efficiently.*” And displaying toolkits on their working desk was beneficial to remind them to use the tools. However, these findings also showed some potential problems, for instance, “*It was not convenient that I must bring the toolkit with me since I always changed my working place from office to home office* (P10, P11).” Therefore, participants suggested that it would be a solution to transfer the on-paper approach into a digital application (i.e., tangible tools for reporting, digital application for overviewing intake data, and easy to check). On the other hand, according to our quantitative results, the using duration with NutriColoring was increased in the home office context, especially during the weekend. The reasons behind this result were identified as “*high engagement in a private working space with less disturbing from others,*” “*more possibilities of coloring the diary while eating,*” and “*no judgement about coloring output from others*” by most participants. This finding gave a potential insight into the context that the relative private and/or individual working space enables to motivate the usage of the NutriColoring toolkit. Future design could investigate different working contexts to develop various healthy eating promotions.

## 6 Discussion

This study presents the design and usefulness test of the NutriColoring toolkit, a Doodling via Coloring approach that aims to prompt self-reporting and self-reflection about daily intake in the working context. NutriColoring toolkit was designed to support healthy eating at work with two design considerations. First, we integrated the Food Pyramid into the NutriColoring toolkit and appropriated food categories with six corresponding colors: Orange (Grains), Green (Vegetables), Yellow (Fruits), Red (Fats, Oils, and sweets), Blue (Milk and Dairy), and Pink (Meat). Second, we explored 30 line-drawing cards with various meal contents to facilitate a color-it-up reporting approach in the working context. A cultural probe study was conducted to understand the user experience of the NutriColoring toolkit compared to the text-based reporting approach (NutriWriting) and test its applicability to workplace healthy eating. We collected quantitative data via NASA-TLX and IMI questionnaires and qualitative interview data with 18 working-age individuals. Our quantitative and qualitative data showed that the NutriColoring toolkit provided users with a positive using experience and motivation in terms of lower frustration and higher enjoyment. The interview results revealed a high acceptance of using the NutriColoring toolkit at work as participants believed that Doodling via Coloring approach could provide freedom for intake reporting exploration and engagement in intake reporting activities in a playful way at work. Our results and findings confirmed our two research questions that the NutriColoring toolkit could be used for self-reporting at work and positively affects self-reflection about personal eating status. Based on these insights, we further derived several design implications for promoting healthy eating during working hours.

### 6.1 Design implications

#### 6.1.1 Simple and interactive self-reporting tools without overburdening

Most participants thought the playful and high-engagement Doodling approach provided by the NutriColoring toolkit design was suitable for relaxation or refreshment in the working context. Based on the colored results display, the NutriColoring toolkit could enable users to self-report daily intake during working hours. Some participants mentioned that the NutriColoring toolkit makes their reporting process occur more frequently because they could easily engage and start the reporting activities spontaneously by coloring a properly illustrated card as a work break. Compared to a text-based journaling tool that requires working-age individuals to record intake repeatedly, NutriColoring based on professional reference (i.e., Food Pyramid) can be easier and more “*work friendly*” to use. Moreover, within the hybrid working context, some participants suggested integrating the self-reporting features of the NutriColoring toolkit into portable digital tools, such as mobile applications or websites, with interactive coloring capabilities. We learned that a digital tool could address issues related to not-at-hand problems due to the switch between the office and the home office, ensuring accessibility. Therefore, future research could explore coloring doodling in digital technologies to make it more adaptive and adjustable. One example could be the use of vision-based sensors, which provide non-intrusive solutions for food monitoring and show promising performance in food recognition, eating behavior detection, intake classification, and food amount estimation (Chen and Kamavuako, 2023).

#### 6.1.2 Challenges and social triggers for motivation and engagement

Various studies have examined the impacts of a game challenge mechanism on promoting healthy eating awareness and behaviors. For instance, Peng (2009) has suggested that using role-playing and interactive tailoring could increase users’ self-reflection on healthy eating as well as their intention to be on a healthy diet. Most participants thought coloring the illustrated cards in the NutriColoring toolkit on each working day enabled them to achieve their personal eating goals step by step unobtrusively. Participants suggested that setting eating goals and daily challenges can positively enhance user experience with paper journaling via the coloring approach, especially the motivation and engagement related to the self-reporting practice. Participants also stated that NutriColoring was easy to learn and performed well with a low learning curve and low mental effort. This simplicity may also lead to boredom and loss of motivation after a few weeks of use. Therefore, for long-term engagement, one possible solution would be to develop the NutriColoring toolkit in a unit social context to facilitate healthy eating via social support. Users in the same workplace with similar eating goals can cooperate or compete via NutriColoring results at work. For example, coworkers with similar eating goals can share coloring results with each other and achieve challenges together; or to protect privacy, users can present their colored results anonymously, and compare personal data with others to stimulate healthy eating patterns safely.

#### 6.1.3 Personalized and artistic achievement

In the NutriColoring toolkit, the research team designed illustrated cards with different meal content in advance. These line-based cards may not correspond to every participant’s daily intake accurately. Most participants thought personalized cards generated by technical system algorithms or designed by the users themselves would increase using motivation and engagement of self-reporting with the NutriColoring toolkit at work. Additionally, by subclassing colored results in a certain period of time (i.e., 1 week, 1 month, and 1 year), participants looked forward to an artistic overview as feedback, which was considered an efficient method to raise a sense of achievement and help users to report intake with Doodling via Coloring approach in the long term.

### 6.2 Limitations and future study

The findings of this study may need to be cautiously interpreted due to the following limitations. First, the sample chosen might have influenced the results of the study. For instance, a study with 18 participants may not be adequate to reveal the impacts of the NutriColoring toolkit on healthy eating promotion in the working context. Our sample mainly consists of participants with little to no experience in creative disciplines, which might differ from the experiences of more experienced individuals. Additionally, we specifically selected participants with a high level of education and those who are not color blind for this study. Therefore, the results may not be representative of the general population when using the NutriColoring toolkit in a working context. Second, our study mainly focused on the usefulness of the NutriColoring toolkit in supporting intake reporting at work for 1 week, while the desirability of the Coloring approach for long-term and everyday use was not evaluated. For our future study, we will upgrade the NutriColoring toolkit and conduct a long-term field study where the Paper Journaling with Coloring approach will be used as an everyday gadget in the working context instead of as a research probe for an experiment. Third, another limitation might be the design aspect. In this study, NutriColoring was integrated into a tangible toolkit with pre-set illustrated cards, which may not accurately reflect individual dietary intake content and consumed food amounts. In future, it will be potential to upgrade the NutriColoring toolkit into digital tools that assist coloring doodling with personalized cards based on individuals’ intake content and amount, offering a more simple and efficient reporting approach.

## 7 Conclusion

This study presents the design and evaluation of the NutriColoring toolkit, a playful self-reporting Doodling with Coloring way for healthy eating at work. In a cultural probe study, we tested the usefulness of the NutriColoring toolkit by comparing it with another traditional food journaling toolkit, NutriWriting. In total, 18 participants were recruited to take part in a 2-week study procedure. The main purposes of this study were to investigate the usefulness of the NutriColoring and the potential effectiveness on self-reflection of intake quality. The quantitative data of NASA-TLX and IMI and the qualitative data of follow-up interviews were collected for analysis. Comparisons between the NutriColoring toolkit and the NutriWriting toolkit showed that participants preferred using NutriColoring for self-reporting of intake in the working context because of its lower frustration, higher enjoyment, competence, and usefulness. Based on the user responses in the follow-up interviews, we found that: First, interactive and portable self-reported tools would be intuitively designed for exploring the flexibility of dietary assessment within the dual contexts between office and home office. Second, establishing dietary objectives and incorporating them into daily goals could elevate the user’s satisfaction when employing a paper journal through a coloring approach. Third, personalization of line-drawing cards is recommended. These three design opportunities will need to be explored for further design of NutriColoring and interactive technologies to promote healthy eating in the working contexts. Besides, to enhance the long-term usage of NutriColoring, it would be beneficial to implement social strategies such as encouraging colleagues in a shared work environment to collaborate in achieving their eating goals together in future.

## Data availability statement

The raw data supporting the conclusions of this article will be made available by the authors, without undue reservation.

## Ethics statement

The studies involving humans were approved by Eindhoven University of Technology. The studies were conducted in accordance with the local legislation and institutional requirements. Written informed consent for participation in this study was provided by the participants’ legal guardians/next of kin.

## Author contributions

SP: Conceptualization, Formal analysis, Investigation, Software, Visualization, Writing – original draft, Methodology. XR: Methodology, Supervision, Validation, Writing – review & editing. SV: Data curation, Methodology, Supervision, Writing – review & editing. AB: Project administration, Supervision, Writing – review & editing.
